# Analgesic Efficacy of Melatonin: A Meta-Analysis of Randomized, Double-Blind, Placebo-Controlled Trials

**DOI:** 10.3390/jcm9051553

**Published:** 2020-05-21

**Authors:** Si Nae Oh, Seung-Kwon Myung, Hyun Jung Jho

**Affiliations:** 1 Department of Family Medicine, Seoul National University Hospital, Seoul 03080, Korea; osinae@hotmail.com; 2 Department of Medicine, Yonsei University Graduate School, Seoul 03722, Korea; 3 Department of Cancer Biomedical Science, National Cancer Center Graduate School of Cancer Science and Policy, Goyang 10408, Korea; 4 Division of Cancer Epidemiology and Management, Research Institute, National Cancer Center, Goyang 10408, Korea; 5 Department of Family Medicine and Center for Cancer Prevention and Detection, Hospital, National Cancer Center, Goyang 10408, Korea; 6 Palliative Care Clinic, Hospital, National Cancer Center, Goyang 10408, Korea; pallmed@ncc.re.kr

**Keywords:** melatonin, pain, analgesia, human, meta-analysis

## Abstract

Previous systematic reviews and meta-analyses of randomized controlled trials have reported controversial findings regarding the effects of melatonin on pain reduction. The aim of this study was to evaluate the efficacy of melatonin on pain among adults using a meta-analysis of randomized, double-blind, placebo-controlled trials (RDBPCTs). PubMed, EMBASE, the Cochrane Library, and the bibliographies of relevant articles were searched up to February 2020. Two of the authors independently evaluated eligibility of the studies based on the pre-determined criteria and extracted data. Standardized mean differences (SMDs) with 95% confidence intervals (CIs) for the pain score change were calculated using a random-effects meta-analysis. Out of 463 that met the initial criteria, a total of 30 trials, which involved 1967 participants with 983 in an intervention group and 984 in a control group, were included in the final analysis. In a random-effects meta-analysis, the use of melatonin reduced chronic pain in all the trials (5 studies, SMD −0.65, 95% CI −0.96 to −0.34, *I*^2^ = 57.2%) and high-quality trials (4 studies, SMD −0.62, 95% CI −1.01 to −0.23, *I*^2^ = 49.3%). Moreover, the use of melatonin significantly reduced acute postoperative pain (11 studies, SMD −0.82, 95% CI −1.40 to −0.25, *I*^2^ = 93.0%). However, the subgroup meta-analysis of high-quality RDBPCTs showed no significant association between them (6 studies, SMD −0.21, 95 % CI −0.66 to 0.24, *I*^2^ = 82.4%). The current study suggests that melatonin might be used in treatment of chronic pain, while there is no sufficient evidence for acute postoperative or procedural pain. Further trials are warranted to confirm its analgesic effect.

## 1. Introduction

Melatonin (N-acetyl-5-methoxytryptamine), a hormone secreted by the pineal gland, affects the regulation of circadian rhythms, sleep, and mood in humans [1]. Various synthetic melatonin preparations, which are widely available at health-food stores and drugstores, have been used for the treatment of sleep disorder [1]. Meanwhile, regarding a low intensity of pain perception during the night, the possible analgesic effect of high melatonin during the night has been proposed as a mechanism [2]. Based on this initial observation, a number of experimental studies in animals have reported the role of melatonin in pain modulation [3].

In humans, melatonin has been evaluated as an analgesic for various types of pain. A qualitative systematic review of a total of eight randomized controlled trials with perioperative melatonin reported inconsistent and limited evidence regarding its analgesic effects [4]. Another systematic review and meta-analysis of a total of eight randomized controlled trials with perioperative melatonin concluded that its analgesic effects were uncertain due to the profound heterogeneity [5]. A recent systematic review and meta-analysis of a total of 19 randomized controlled trials with the use of melatonin for various types of pain reported a significant reduction of pain [5,6]. The study, however, included open-label trials and active-control trials, and had not performed subgroup analyses by important factors such as methodological quality. Further, additional randomized controlled trials have been published since, and have reported inconsistent findings on the analgesic effect of melatonin.

The aim of the current study was to evaluate the efficacy of melatonin on pain among adults using a meta-analysis of randomized, double-blind, placebo-controlled trials (RDBPCTs).

## 2. Materials and Methods

### 2.1. Data Sources and Searches

The systematic review and meta-analysis was performed according to the Preferred Reporting Items for Systematic Reviews and Meta-analyses (PRISMA) statement [7]. We searched PubMed, EMBASE, and the Cochrane Library using keywords in 11 February 2020. The keywords were as follows: “melatonin” and “pain”. The bibliographies of relevant articles were reviewed to locate additional publications from the previous review articles and reference lists.

### 2.2. Study Selection and Eligibility

We included RDBPCTs that investigated the effect of melatonin in all types of pain in adults and reported preintervention and postintervention quantitative data. In the current meta-analysis, we excluded open-label trials such as randomized controlled trials that did not use placebos as a control group. Two authors (S.N.O. and S.K.M.) assessed the eligibility of studies by the pre-determined selection criteria. Discrepancies were resolved by discussion.

### 2.3. Assessment of Methodological Quality

Two authors (S.N.O. and H.J.C.) assessed the methodological quality of RDBPCTs, and disagreements were resolved by consensus in discussion with a third reviewer (S.K.M). We assessed the risk of bias on the basis of Cochrane Risk of Bias Tool [8]. Trials that had a low risk of bias in more than the average number of items in all the trials were considered to have an overall low risk of bias in this study.

### 2.4. Main and Subgroup Analysis

For the main analysis, we examined the associations between the use of melatonin and the pain score changes as well as those between the use of melatonin and the changes in analgesic consumption. Moreover, subgroup meta-analyses for each outcome were performed according to various factors as follows: pain type (acute procedural pain, acute postoperative pain—local and epidural anesthesia, acute postoperative pain—general anesthesia, chronic pain—defined as pain lasting three months or longer at the time of enrollment, and pain duration not reported) and methodological quality score (number of low risk of bias <6 and ≥6). Furthermore, we extracted data on adverse events.

### 2.5. Statistical analyses

We calculated pooled standardized mean differences (SMDs) with their corresponding 95% confidence intervals (CIs). A random-effects model meta-analysis based on the Der Simonian and Laird method was used in the current study because individual trials were carried out in the different populations. We transformed median (interquartile range or range) values to mean (standard deviation) values [8]. If more than two doses of melatonin in a trial were used, the dose which was closer to the average dose of all trials was chosen. If a scale decreases with pain intensity, we multiplied the mean values from one set of studies by −1 to make all the scales point in the same direction [8]. For the test of heterogeneity across studies, Higgins *I*^2^ was used to measure the percentage of total variation across publications [9]. An *I*^2^ value greater than 50% was regarded as substantial heterogeneity [9]. We constructed a funnel plot with 1/(SE), a measure of sample size, plotted against effect size, to examine publication bias [10]. (Supplementary Figure S1) The statistical analysis was performed using Stata SE version 13.1 software package (StataCorp, College Station, TX, USA).

## 3. Results

### 3.1. Identification of Relevant Studies

**Figure 1:**
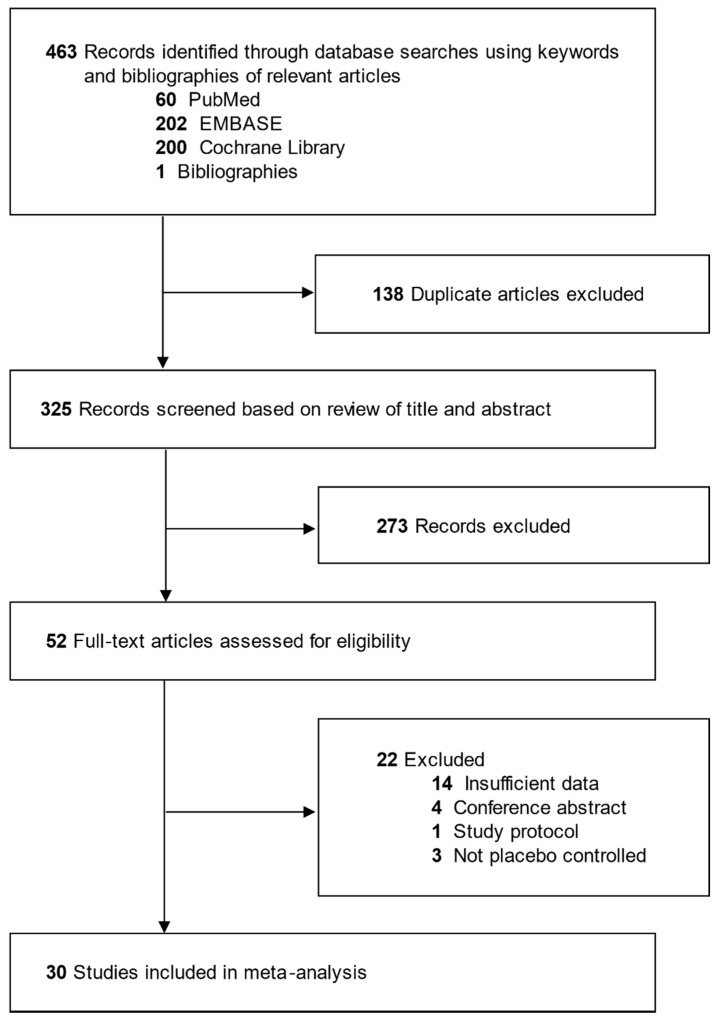
Diagram of the study selection process.

Figure 1 shows a flow diagram for the study selection process. A total of 463 articles were identified by the initial search of four databases and hand-searching relevant bibliographies. After excluding 138 duplicated articles, two of the authors independently evaluated eligibility of all studies and excluded an additional 273 articles that did not meet the pre-determined selection criteria depending on the title and abstract of each article. Among them, 22 articles were excluded after reviewing the full texts of the remaining 52 articles for the following reasons: insufficient data (*n* = 14); conference abstract (*n* = 4); study protocol (*n* = 1); and not placebo controlled (*n* = 3). The remaining 30 RDBPCT were included in the final analysis [11,12,13,14,15,16,17,18,19,20,21,22,23,24,25,26,27,28,29,30,31,32,33,34,35,36,37,38,39,40].

### 3.2. General Characteristics of Trials

Table 1 shows the general characteristics of 30 RDBPCT included in the final analysis. The included trials were published between 2006 and 2019, and they involved a total of 1967 participants (983 in an intervention group and 984 in a control group). Out of 30 trials, 12 trials investigated acute postoperative pain after the surgery under general anesthesia [12,13,14,15,18,19,20,24,26,30,36,40], four trials did acute postoperative pain after the surgery under local and epidural anesthesia [15,25,28,29], four trials investigated acute procedural pain [23,27,32,33], and five trials investigated chronic pain [17,21,35,37,38]. The duration of pain was not reported in the remaining five trials [11,22,31,34,39]. In the methodological quality score assessed by the Cochrane Risk of Bias Tool, the average number of low risk of bias was 5.8, and 19 trials demonstrated low risk of bias in 6 items or more and were considered to be high quality [14,16,18,19,20,21,22,24,28,29,31,32,33,34,35,36,37,38,39], while the remaining 11 trials demonstrated a low risk of bias in 5 items or less [11,12,13,15,17,23,25,26,27,30,40] (Table 2).

### 3.3. Association between the Use of Melatonin and Pain Score Changes

**Figure 2:**
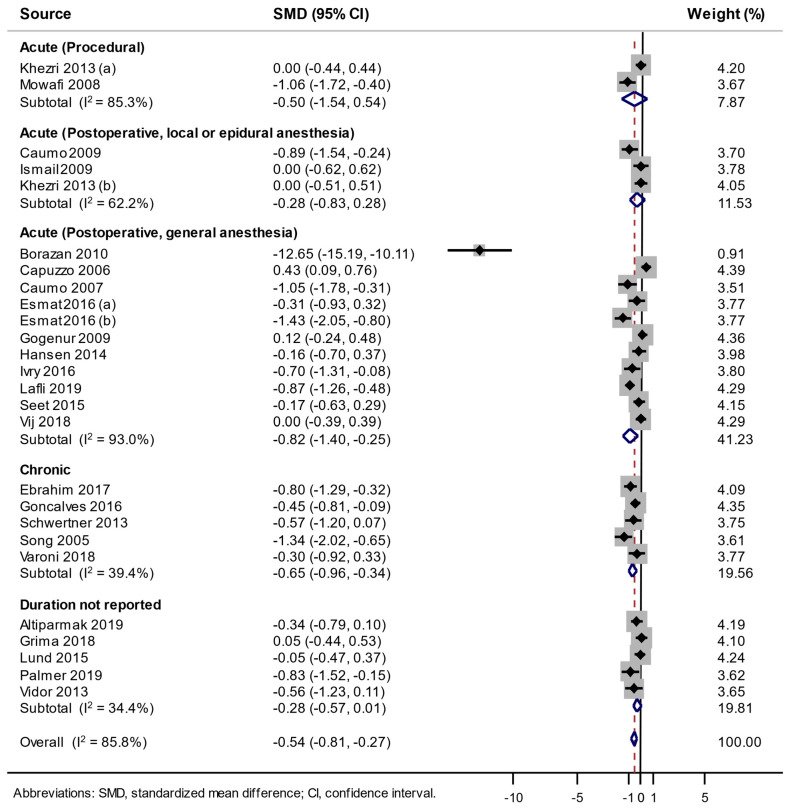
Use of melatonin and change of pain score in a random-effects meta-analysis of randomized, double-blind, placebo-controlled trials.

As shown in Figure 2, a random-effects meta-analysis of a total of 26 RDBPCTs showed that the use of melatonin significantly decreased pain scores compared with a placebo with substantial heterogeneity (SMD - 0.54, 95% CI −0.81 to −0.27, *I*^2^ = 85.8%, *n* = 26). In the subgroup meta-analysis by pain type, the use of melatonin decreased pain scores in acute postoperative pain after the surgery under general anesthesia (SMD −0.82, 95% CI −1.40 to −0.25, *I*^2^ = 93.0%, *n* = 11), and in chronic pain (SMD −0.65, 95% CI −0.96 to −0.34, *I*^2^ = 39.4%, *n* = 5).

However, in the subgroup meta-analysis for the acute postoperative pain after surgery under general anesthesia by number of items of low risk of bias, although a significantly large pain reduction with the use of melatonin was observed in those with low risk of bias in less than six items (SMD −2.13, 95% CI −3.46 to −0.81, *I*^2^ = 96.0%, *n* = 5), there was no significant pain reduction in the trials with low risk of bias in six or more items. Meanwhile, in the subgroup meta-analysis for the chronic pain by number of items of low risk of bias, melatonin was effective for reducing pain intensity in both the trials with low risk of bias in six or more items (SMD −0.62, 95% CI −1.01 to −0.23, *I*^2^ = 49.3%, *n* = 4) and those with low risk of bias in less than six items (SMD −0.80, 95% CI −1.29 to −0.32, *I*^2^ = not applicable, *n* = 1) (Table 3).

### 3.4. Association between the Use of Melatonin and Changes in Analgesic Consumption

**Figure 3:**
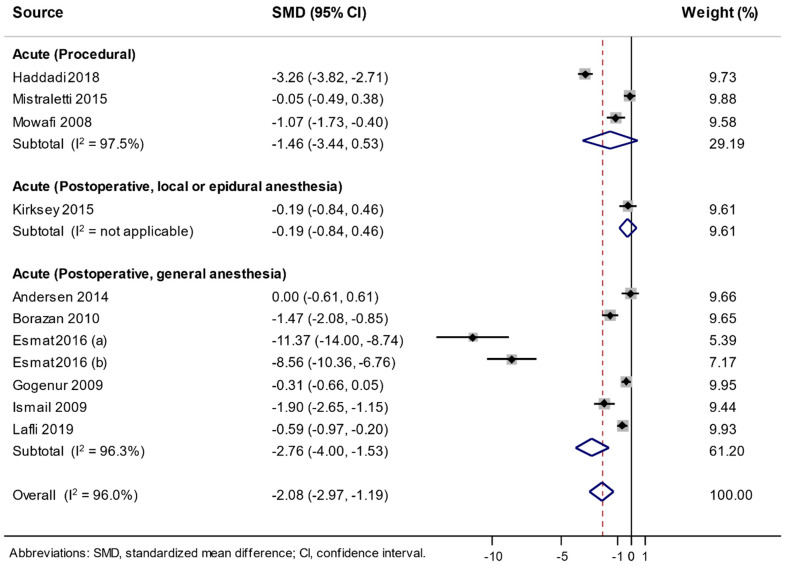
Use of melatonin and change of analgesic consumption in a random-effects meta-analysis of randomized, double-blind, placebo-controlled trials.

As shown in Figure 3, a random effect meta-analysis of a total of 11 RDBPCTs showed that the use of melatonin significantly decreased analgesic consumption compared with a placebo with substantial heterogeneity (SMD −2.08, 95% CI −2.97 to −1.19, *I*^2^ = 96.0%, *n* = 11). In the subgroup meta-analysis by pain type, the use of melatonin decreased analgesic consumption in acute postoperative pain after the surgery under general anesthesia (SMD −2.76, 95% CI −4.00 to −1.53, *I*^2^ = 96.3%, *n* = 7), while there was no significant reduction in analgesic consumption in the trials with acute procedural pain and acute postoperative pain after the surgery under local or epidural anesthesia.

Furthermore, in the subgroup meta-analysis for the acute postoperative pain after the surgery under general anesthesia by number of items of low risk of bias, there was a significant reduction of analgesic consumption in both the trials with low risk of bias in six or more items (SMD −4.68, 95% CI −7.55 to −1.81, *I*^2^ = 97.9%, *n* = 4) and those with low risk of bias in less than six items (SMD −1.27, 95% CI −2.09 to −0.46, *I*^2^ = 83.6%, *n* = 3) (Table 4).

### 3.5. Adverse Events

Fifteen studies out of 30 studies assessed adverse events [12,17,18,19,21,22,24,25,28,29,31,32,33,38,39]. Dizziness [18,19,25,29,33] and drowsiness [17,18,19,33] were found in five studies and four studies, respectively. One study reported fatigue [17], and another reported mild headaches [26,28]. The remaining eight studies reported no serious adverse events [12,21,22,24,31,32,38,39].

## 4. Discussion

### 4.1. Summary of Findings

In the current meta-analysis of 30 RDBPCTs, we found that the use of melatonin was associated with the improvement of chronic pain regardless of study quality, specifically in endometriosis, irritable bowel syndrome, and migraines. There was no sufficient evidence that the use of melatonin has beneficial effects for acute postoperative or procedural pain. Although there were effects on pain intensity in acute postoperative pain after surgery under general anesthesia in low-quality studies, there was no effect for it in high-quality studies. Meanwhile, there was an effect on analgesic consumption in acute postoperative pain after surgery under general anesthesia in both high-quality studies and low-quality studies. For acute procedural pain and acute postoperative pain after the surgery under local or epidural anesthesia, there were too few studies to determine its efficacy.

### 4.2. Possible Mechanisms for Analgesic Effects of Melatonin

There are several possible biological mechanisms for the analgesic effect of melatonin. Previous experimental studies in animals and humans have reported its analgesic effect in various nociceptive and neuropathic pain models. In rodents, melatonin has shown antinociceptive, antihyperalgesic, and antiallodynic effects against various noxious stimuli, inflammation, and nerve injury [41]. One of the most important mechanisms of action is an activation of melatonin receptors, termed MT1 and MT2, distributed in important regions in pain control, such as lamina I-V and X of the spinal cord, thalamus, hypothalamus, spinal trigeminal tract, and trigeminal nucleus [42,43,44]. Activation of melatonin receptors leads to a Gi-protein-mediated decrease of cyclic AMP levels and inhibits Ca2+ channels so that intracellular Ca2+ levels decrease [45]. An increase in intracellular Ca2+ levels beyond a certain threshold has been known to be critical in central sensitization associated with inflammatory and neuropathic pain [46]. The activation of melatonin receptors also activates K+ channels which inhibit an action potential firing in neurons [47]. Several other second messenger molecules like cGMP, diacylglycerol, inositol triphosphate, and arachidonic acid are regulated by melatonin receptors [48]. Melatonin indirectly interacts with other receptor systems including benzodiazepinergic, opioidergic, serotonergic, dopaminergic, adrenergic, glutaminergic, and NO-cyclic GMP-PKG signaling pathway [49].

Melatonin also has anti-inflammatory and antioxidative effects which may affect peripheral nociception and hyperalgesia by reducing inflammation and tissue damage [3]. It might directly interact with specific binding sites in lymphocytes and macrophages and inhibit the production of pro-inflammatory cytokines [50,51]. Melatonin is also a direct free radical scavenger which neutralizes a number of free radicals including reactive oxygen and nitrogen species [52]. It also stimulates antioxidative enzymes like glutathione peroxidase, glutathione reductase, and superoxide dismutase [52].

Moreover, a few human experimental studies have been reported. Stefani et al. reported dose-dependent analgesic effects of melatonin in healthy volunteers [53]. A single dose of sublingual melatonin 0.15 mg/kg and 0.25 mg/kg showed a significant increase in pressure and heat pain threshold and tolerance, and there was a correlation between serum melatonin concentrations and changes in pain threshold and tolerance [53]. However, in a burn injury model in healthy volunteers, Anderson et al. showed no analgesic, antihyperalgesic, or anti-inflammatory effects of intravenous melatonin 10 mg and 100 mg comparing to placebos [54].

### 4.3. Comparisons with Previous Studies and Strengths of Our Study

In the meantime, previous RDBPCTs have reported inconsistent findings on the analgesic effects of melatonin in various types of pain. Since 2010, several systematic reviews and meta-analyses have been published on this topic. In 2010, a systematic review of randomized trials without meta-analysis reported that five studies showed an opioid-sparing effect or reduced perioperative pain scores, whereas three studies were contradictory [4]. In 2014, a meta-analysis of eight randomized trials reported an analgesic effect of melatonin in postoperative pain [5]. However, the authors concluded that the magnitude of effect was unreliable because of substantial heterogeneity.

A recent meta-analysis of 19 randomized trials published in 2017 showed that melatonin significantly reduced the pain intensity indicated by pain scores in the overall anti-nociception effect and in the subgroup meta-analyses by operation-associated pain, inflammatory pain, and procedural pain [6]. However, it included trials without a placebo control group and assessed the quality of trials using only the Jadad scale, which has been criticized and explicitly discouraged by the Cochrane reviews because of issues with the generic problems of scales, its strong emphasis on reporting rather than on conduct, and it does not cover one of the most important potential biases in randomized trials, i.e., allocation concealment [9].

Unlike these systematic reviews and meta-analyses, we performed subgroup meta-analyses according to various factors including the risk of bias by the Cochrane Risk of Bias Tool [8]. Interestingly, the trials with low risk of bias in six or more items, which are considered as having high quality, showed no significant reduction of pain scores in acute postoperative pain after surgery under general anesthesia, whereas those with low risk of bias in less than 6 items, which are considered as having low quality, showed a large reduction of pain scores. Cochrane reviews explicitly discourage the use of scales or scores for assessing quality or risk of bias because of difficulties in justification of assigning weights to different items in the scale regarding the calculation of a summary score and unreliable assessments of validity by the scales [8]. Despite these limitations, we think that it would be helpful to know if there is any tendency in the study findings according to study quality or bias. Based on the findings from the subgroup meta-analyses by the number of items of low risk of bias, we suggest that melatonin might have no significant effect on acute postoperative pain after surgery under general anesthesia because trials with low risk of bias in six or more items are more likely to show the results closer to the truth than those with low risk of bias in less than six items.

### 4.4. Possible Reasons for No Analgesic Effect of Melationin in Acute Postoperative or Procedural Pain

There are possible explanations or reasons why no analgesic effect of melatonin was shown in acute postoperative or procedural pain. First, the optimal timing of melatonin administration is not established yet. In most RDBPCTs for perioperative or procedural pain included in the current study, melatonin was administered once orally 60–90 min before the surgery or procedure. Time to maximal plasma concentration is approximately 50 min following oral formulation of melatonin, and elimination half-life is 45 min [55]. Therefore, it might not be appropriate to administer melatonin preoperatively in order to reduce postoperative pain. Second, melatonin might not have a strong analgesic effect compared to other proven analgesics, such as opioids and nonsteroidal anti-inflammatory drugs (NSAIDs). In most trials, the study subjects received those opioids or NSAIDs in both the melatonin and control groups. Thus, melatonin administration as a premedication might not show any additional analgesic effects. Last, melatonin might be ineffective for perioperative or procedural pain. Our findings showed that melatonin was effective for chronic pain. It might be related to the differences in pathophysiology between acute nociceptive pain, such as acute postoperative or procedural pain, and chronic pain [56,57].

### 4.5. Limitations

Our study has several limitations. First, we included only five studies including 309 patients for chronic pain. Therefore, further large trials are warranted to confirm our findings on the analgesic effect of exogenous melatonin for chronic pain. Second, 10 out of the 26 RDBPCTs included in the current meta-analysis were not designed specifically to investigate the analgesic effect of melatonin as the primary endpoint. In general, findings in the secondary endpoint might be due to chance because the design of the trial is not specifically powered to assess it. Last, we estimated means and standard deviations from median and interquartile ranges or ranges in ten RDBPCTs. If data are skewed, they might not be estimated accurately [8].

## 5. Conclusions

In summary, melatonin might be used in the treatment of chronic pain, specifically in endometriosis, irritable bowel syndrome, and migraines. There was no sufficient evidence to support the use of melatonin for acute postoperative or procedural pain based on the meta-analysis of high-quality RDBPCTs. However, further trials are warranted to confirm its analgesic effects. Particularly, high methodological quality research is needed regarding acute postoperative pain after surgery under general anesthesia.

## Figures and Tables

**Table 1:** General characteristics of randomized, double-blind, placebo-controlled trials included in the final analysis.

Source	Country	Participants (Mean Age, y; Women, %)	Intervention vs. Control	Duration of Medication	Scoring Instrument (Scale); Endpoint	Change in Pain Score (SD)	
						Intervention Group	Placebo Group
Altiparmak 2019 [11]	Turkey	80 patients with neuropathic pain (49.3; 48.8)	Melatonin 3 mg PO + gabapentin 900 mg daily vs.placebo + gabapentin 900 mg daily	30 days	VRS (0–10); secondary	−4.1 (1.3)	−3.7 (1.3)
Anderson 2014 [12]	Denmark	40 patients undergoing laparoscopic cholecystectomy (45; 100)	Melatonin 10 mg IV vs. placebo	At the time of surgical incision	NA	NA	NA
Borazan 2010 [13]	Turkey	52 patients undergoing open prostatectomy (57; 0)	Melatonin 6 mg PO vs. placebo	The night before and 60 min before surgery	VAS (0–100); secondary	27 (1.0)	47 (2.0)
Capuzzo 2006 [14]	Italy	150 patients undergoing surgery (73.2, 52)	Melatonin 10 mg PO vs. placebo	90 min before surgery	VAS (0–10); secondary	2 (3.0)	1 (1.5)
Caumo 2007 [15]	Brazil	35 patients undergoing total abdominal hysterectomy (44.8, 100)	Melatonin 5 mg PO vs. placebo	The night before and 60 min before surgery	VAS (0–100); primary	30 (16.5)	47 (16.0)
Caumo 2009 [16]	Brazil	63 patients undergoing total abdominal hysterectomy (43.4, 100)	Melatonin 5 mg PO vs. clonidine 100 ug PO vs. placebo	The night before and 60 min before surgery	VAS (0–100); primary	30 (22.4)	50 (22.4)
Ebrahimi 2017 [17]	Iran	105 patients with migraine (38.9, 49)	Melatonin 3 mg PO daily vs. Sodium valproate 200 mg vs. placebo	8 weeks	VAS (0–10); primary	−3.8 (2.8)	−1.3 (3.4)
Esmat 2016 [18]	Egypt	75 patients undergoing lumbar laminectomy (44.6, 48)	Melatonin 7 mg/8 h patch vs. Fentanyl 50 ug/h patch vs. placebo	From 2 h before surgery to 12 h after surgery	VAS (0–10); primary	3 (0.7)	4 (0.7)
Esmat 2016 [19]	Egypt	62 patients undergoing laparoscopic cholecystectomy (44.3, 100)	Melatonin 7 mg/8 h patch vs. Nicotine 15 mg/16 h patch vs. placebo	From 2 h before surgery to 12 h after surgery	VAS (0–10); primary	4 (0.7)	4.5 (2.2)
Gogenur 2009 [20]	Denmark	136 patients undergoing laparoscopic cholecystectomy (44, 71)	Melatonin 5 mg PO daily vs. placebo	3 days	VAS (0–100); secondary	51 (24.5)	48 (25.0)
Gonçalves 2016 [21]	Brazil	196 patients with migraine (36.9; 44)	Melatonin 3 mg PO daily vs. Amitriptyline 25 mg vs. placebo	12 weeks	NRS (0–10); secondary	−3.5 (3.95)	−1.8 (3.61)
Grima 2018 [22]	Australia	66 patients with traumatic brain injury (37; 33)	Melatonin 2 mg PO daily vs. placebo	4 weeks	SF-36 (0–100); secondary	2.07 (17.64)	1.27 (17.64)
Haddadi 2018 [23]	Iran	180 patients undergoing retrobulbar eye block for cataract surgery (63.6; 56)	Melatonin 6 mg PO vs. placebo	60 min before surgery	NA	NA	NA
Hansen 2014 [24]	Denmark	54 patients undergoing breast cancer surgery (51, 100)	Melatonin 6 mg PO daily vs. placebo	From 1 week before surgery to 12 weeks after surgery	VAS (0–100); secondary	97 (100.7)	130 (277.8)
Ismail 2009 [25]	Saudi Arabia	40 patients undergoing cataract surgery (72.8, 48)	Melatonin 10 mg PO vs. placebo	90 min before surgery	VAS (0–100); primary	30 (14.8)	30 (11.1)
Ivry 2016 [26]	Israel	60 patients undergoing bariatric surgery (43, 46)	Melatonin 5 mg PO vs. placebo	Night before and 2 h before surgery	QoR-15 (0–10); primary	3.9 (5.4)	7.5 (4.9)
Khezri 2013 [27]	Iran	120 patients undergoing retrobulbar eye block for cataract surgery (73; 38)	Melatonin 6 mg PO vs. gabapentin 600 mg PO vs. placebo	90 min before surgery	VPS (0–10); primary	4.0 (5.9)	4.0 (6.3)
Khezri 2013 [28]	Iran	60 patients undergoing cataract surgery (63.5; 60)	Melatonin 3 mg SL vs. placebo	60 min before surgery	VPS (0–100); secondary	10.0 (7.4)	10.0 (7.4)
Kirksey 2015 [29]	United States	37 patients undergoing total knee arthroplasty (70; 73.7)	Melatonin 5 mg PO vs. placebo	6 days	NA	NA	NA
Laflı 2019 [30]	Turkey	165 patients undergoing major abdominal surgery (47.3; 75)	Melatonin 6 mg PO vs. Vitamin C 2 g PO vs. Placebo	1 h before surgery	VAS (0–10); primary	3.04 (1.82)	4.75 (2.09)
Lund 2015 [31]	Denmark	72 patients with advanced cancer (64; 66)	Melatonin 20 mg PO daily vs. placebo	15 days	EORTC QLQ-C15-PAL (0–100); secondary	0.8 (19.3)	1.9 (22.2)
Mistraletti 2015 [32]	Italy	82 critically ill patients requiring invasive or non-invasive respiratory assistance	Melatonin 6 mg PO daily vs. placebo	From the third intensive care unit (ICU) day to ICU discharge	NA	NA	NA
Mowafi 2008 [33]	Saudi Arabia	40 patients with tourniquet-related pain for hand surgery (44.6; 45)	Melatonin 10 mg PO vs. placebo	90 min before surgery	VPS (0–100); primary	30.0 (7.4)	40.0 (11.1)
Palmer 2019 [34]	Brazil	36 patients with breast cancer receiving adjuvant chemotherapy (54.2; 100)	Melatonin 20 mg PO daily vs. placebo	10 days	NRS (0–10); primary	−3.25 (1.16)	−1.91 (1.60)
Schwertner 2013 [35]	Brazil	40 patients with endometriosis (36.8; 100)	Melatonin 10 mg PO daily vs. placebo	8 weeks	VAS (0–10); primary	−3.08 (3.62)	−1.16 (3.13)
Seet 2015 [36]	Singapore	76 patients undergoing all four third molar teeth extraction (22.7; 33)	Melatonin 6 mg PO vs. placebo	90 min before surgery	VAS (0–100); primary	11.3 (11.0)	13.2 (11.0)
Song 2005 [37]	Singapore	42 patients with irritable bowel syndrome (27.2; 60)	Melatonin 3 mg PO daily vs. placebo	2 weeks	NRS (0–10); primary	−2.35 (1.34)	−0.70 (1.12)
Varoni 2018 [38]	Italy	20 patients with burning mouth syndrome (64.4; 80)	Melatonin 12 mg PO daily	8 weeks	VAS (0–10); Primary	0.6 (2.2)	1.2 (1.8)
Vidor 2013 [39]	Brazil	32 patients with temporomandibular disorders (32.3; NR)	Melatonin 5 mg PO daily vs. placebo	4 weeks	VAS (0–10); primary	−2.55 (2.96)	−0.91 (2.92)
Vij 2018 [40]	India	100 patients undergoing laparoscopic cholecystectomy (42.8; 74)	Melatonin 5 mg PO daily vs. Placebo	3 days	VAS (0–100); Secondary	30.0 (12.5)	30.0 (15.8)

Abbreviation: FLACC, the face, legs, activity, cry, consolability scale; EORTC QLQ-C15-PAL, European organization for research and treatment of cancer quality of life questionnaire core 15 palliative version; FPS, faces pain scale; NR, not reported; NRS, numeric rating scale; QoR-15, quality of recovery 15 questionnaire score; SD, standard deviation; VAS, visual analog scale; VPS, verbal pain score; VRS, verbal rating scale.

**Table 2:** Summary of risk of bias assessment for randomized, double-blind, placebo-controlled trials. ^(a)^

Source	Random Sequence Generation	Allocation Concealment	Blinding of Participants, and Personnel	Blinding of Outcome Assessment	Incomplete Outcome Data	Selective Reporting	Other Bias	No. of Low Risk of Bias
Altiparmak 2019 [11]	low	unclear	unclear	unclear	low	low	low	4
Anderson 2014 [12]	low	unclear	unclear	unclear	low	low	low	4
Borazan 2010 [13]	low	unclear	unclear	low	low	low	low	5
Capuzzo 2006 [14]	low	low	low	low	low	low	low	7
Caumo 2007 [15]	low	unclear	unclear	low	low	low	low	5
Caumo 2009 [16]	low	low	unclear	low	low	low	low	6
Ebrahimi 2017 [17]	low	low	unclear	low	unclear	low	low	5
Esmat 2016 [18]	low	low	unclear	low	low	low	low	6
Esmat 2016 [19]	low	low	unclear	low	low	low	low	6
Gögenur 2009 [20]	low	low	low	low	low	low	low	7
Gonçalves 2016 [21]	low	low	low	low	low	low	low	7
Grima 2018 [22]	unclear	low	low	low	low	low	low	6
Haddadi 2018 [23]	unclear	unclear	unclear	unclear	low	low	low	3
Hansen 2014 [24]	low	low	low	low	low	low	low	7
Ismail 2009 [25]	low	unclear	high	low	low	low	low	5
Ivry 2016 [26]	low	unclear	unclear	low	low	low	low	5
Khezri 2013 [27]	low	low	unclear	low	low	low	low	6
Khezri 2013 [28]	low	unclear	unclear	low	low	low	low	5
Kirksey 2015 [29]	unclear	low	low	low	low	low	low	6
Laflı 2019 [30]	low	unclear	unclear	low	low	low	low	5
Lund 2015 [31]	low	low	low	low	low	low	low	7
Mistraletti 2015 [32]	low	low	low	low	low	low	low	7
Mowafi 2008 [33]	low	low	unclear	low	low	low	low	6
Palmer 2019 [34]	low	low	low	low	low	low	low	7
Schwertner 2013 [35]	low	low	low	low	low	low	low	7
Seet 2015 [36]	low	low	low	low	low	low	low	7
Song 2005 [37]	unclear	low	low	low	low	low	low	6
Varoni 2018 [38]	low	unclear	low	low	low	low	low	6
Vidor 2013 [39]	low	low	low	low	low	low	low	7
Vij 2018 [40]	low	unclear	unclear	unclear	unclear	low	low	3

^(a)^ Based on the Cochrane Risk of Bias Tool.

**Table 3:** Use of melatonin and pain score change in the subgroup meta-analysis of randomized, double-blind, placebo-controlled trials.

Type of Pain	No. of Low Risk of Bias	No. of Trials	Summary SMD (95% CI)	Heterogeneity, *I*^2^ (%)
Acute pain—postoperative, local or epidural anesthesia	≥ 6	1 [15]	−0.893 (−1.544, −0.241)	NA
	< 6	2 [23,26]	0.000 (−0.392, 0.392)	0
Acute pain—postoperative, general anesthesia	≥ 6	6 [13,17,18,19,22,32]	−0.212 (−0.664, 0.240)	82.4
	< 6	5 [12,14,24,27,36]	−2.134 (−3.456, −0.812)	96.0
Chronic pain	≥ 6	4 [20,31,33,34]	−0.618 (−1.011, −0.225)	49.3
	< 6	1 [16]	−0.803 (−1.291, −0.316)	NA

Abbreviations: NA, not applicable; SMD, standardized mean difference; CI, confidence interval.

**Table 4:** Use of melatonin and analgesic consumption change in the subgroup meta-analysis of randomized, double-blind, placebo-controlled trials.

Type of Pain	No. of Low Risk of Bias	No. of Trials	Summary SMD (95% CI)	Heterogeneity, *I*^2^ (%)
Acute pain—procedural	≥ 6	2 [32,33]	−0.527 (−1.517, 0.464)	84.0
	< 6	1 [23]	−3.262 (−3.818, −2.706)	NA
Acute pain—postoperative, general anesthesia	≥ 6	4 [12,18,19,20]	−4.676 (−7.546, −1.806)	97.9
	< 6	3 [13,25,30]	−1.271 (−2.087, −0.455)	83.6

Abbreviation: NA, not applicable; SMD, standardized mean difference; CI, confidence interval.

## Supplementary Materials

The following are available online at https://www.mdpi.com/2077-0383/9/5/1553/s1, Figure S1: Publication bias analysis.
