# Supplementary figures and images for: Analgesic Efficacy of Melatonin: A Meta-Analysis of Randomized, Double-Blind, Placebo-Controlled Trials
Source: J Clin Med. 2020 May 21;9(5):1553. doi: 10.3390/jcm9051553 (PMC7291209; doi:10.3390/jcm9051553)

**Supplementary Figure S1. Publication bias analysis**

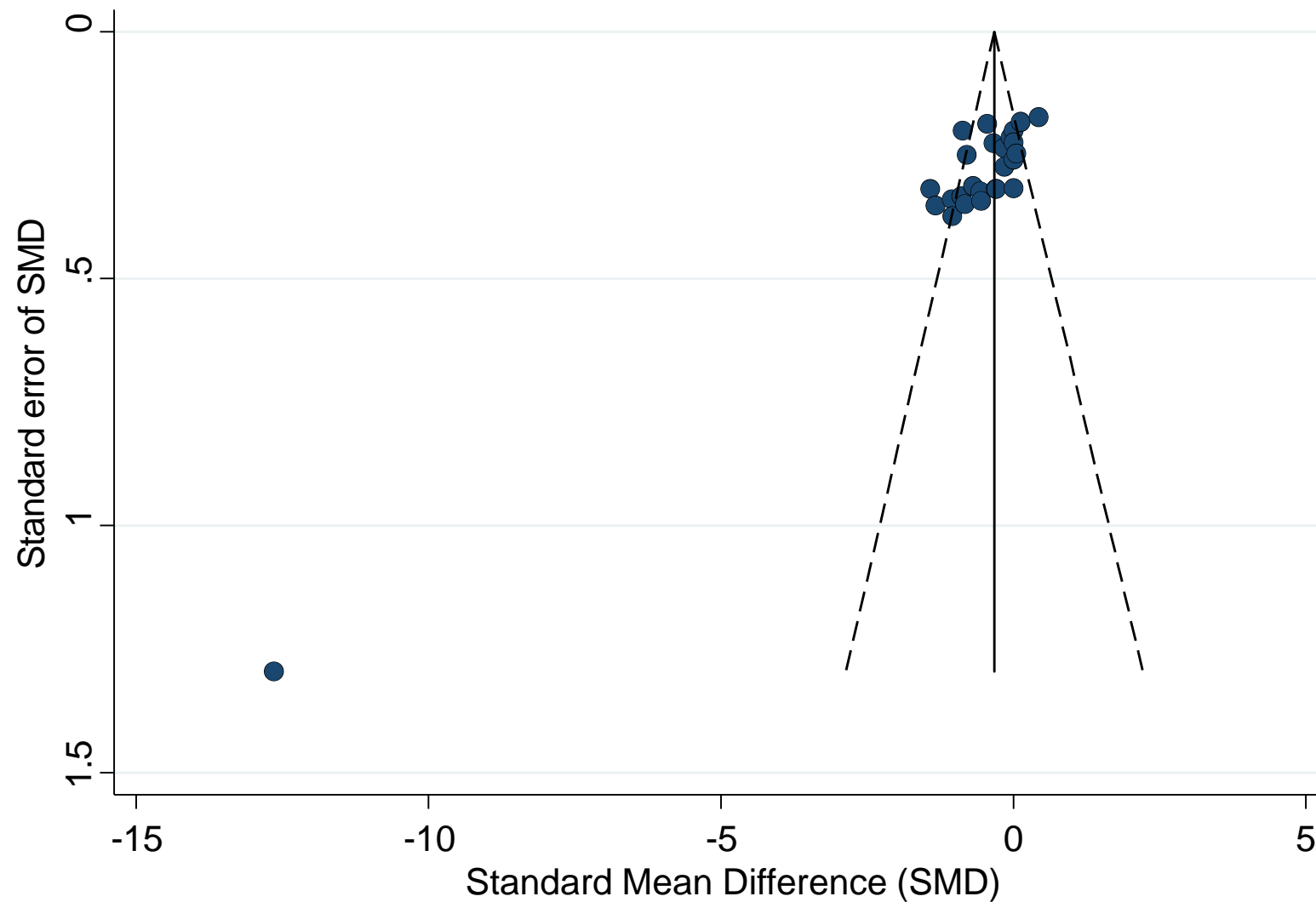

Supplement: Supplementary file 1 [file jcm-09-01553-s001.pdf]
